# Genetic recombination among tomato yellow leaf curl virus isolates in commercial tomato crops in Kuwait drives emergence of virus diversity: a comparative genomic analysis

**DOI:** 10.1186/s13104-023-06319-w

**Published:** 2023-05-07

**Authors:** Ebtisam Al-Ali, Hanadi Al-Hashash, Abrar Akbar, Hamed Al-Aqeel, Nabila Al-Shayji, Mohammed Alotaibi, Ahmed Ben Hejji

**Affiliations:** Kuwait Institute for Scientific Research, Environmental and Life Science Research Center, Biotechnology Program, 13109 Safat, Kuwait

**Keywords:** Begomoviruses, Molecular genomics, Genetic diversity, Genetic recombination, Tomato viruses

## Abstract

**Objective:**

Whitefly-transmitted tomato yellow leaf curl virus (TYLCV) continues to be a major constraint to tomato production in Kuwait. However, very limited information is available about the population structure and genetic diversity of TYLCV infecting tomato in Kuwait.

**Results:**

Whole genome sequences of 31 isolates of TYLCV, collected from commercial tomato crops grown in northern (Abdally) and southern (Al Wafra) parts of Kuwait, were deciphered. Eighteen isolates of TYLCV are identified as potential genetic recombinants. The isolates Abdally 6A and Abdally 3B reported in this study were identified to be potential recombinants. Compared to the 15 isolates from the Abdally area, and the three previously reported KISR isolates of Kuwait, six out of sixteen Al Wafra isolates showed an insertion of 19 extra nucleotides near the 5′-end. There are also four nucleotide variations before the 19-extra-nucleotides. The additional 19 nucleotides observed in nine isolates indicate that these isolates might have resulted from a single gene recombination/insertion event. Molecular phylogeny based on complete genome sequences of TYLCV isolates suggests transboundary movement of virus isolates due to geographic proximity. The information presented herein is quite useful for the comprehension of TYLCV biology, epidemiology and would aid in the management of disease in the long run.

## Introduction

Tomato yellow leaf curl virus (TYLCV) (Genus *Begomovirus*, Family *Geminiviridae*) [1], causes significant damages to tomato in both quantitative and qualitative terms. Symptoms associated with TYLCV infection in tomato include chlorotic leaf edges, upward leaf cupping, leaf mottling, reduced leaf size, and flower drop. Symptoms on the fruit make the agricultural product unmarketable. Thus, TYLCV can inflict a severe impact on tomato production. Tomato plants infected at an early stage won't bear fruit and plants are severely stunted. In early virus infections, 100% crop loss is not uncommon. The virus is generally transmitted by whitefly, *Bemisia tabaci*, and the vector has developed resistance to many of the insecticides, which limits the vector control option [2–6].

The virion of TYLCV is composed of twin icosahedral capsids. The viral genome consists of a single-stranded circular DNA genome of about 2.7 kb, which encodes six open reading frames (ORFs), two (V1 and V2) in viral sense and four (C1- C4) in complementary sense. All the proteins encoded by begomoviruses are multifunctional. Protein encoded by ORF V1 is involved in encapsidation of the genome into geminate particles, insect transmission and long distant movement. Protein derived from V2 ORF plays a role in cell-to-cell movement and suppression of host-mediated RNA silencing. C1 protein is involved in viral DNA replication, while ORF C2-derived protein activates viral DNA transcription and is also a host RNA silencing suppressor. Protein encoded by ORF C3 plays a role in enhancing viral DNA replication, and ORF C4 encoded protein is known to be a RNA silencing suppressor [7, 8]. Besides these ORFs, the TYLCV genome has a 300-nucleotide (nt) non-coding intergenic region (IR), which possesses a bidirectional promoter and a core conserved nona-nucleotide sequence (TAATATTAC). The nona-nucleotide sequence is recognized by the C1 protein, and it serves as the origin of replication. A great deal of information was generated over the years about the diversity, epidemiology and molecular biology of the disease and the virus [9–11].

Management of viral diseases, especially those caused by TYLCV, is challenging and expensive. The whitefly vector has a wide host range, and several weeds serve as reservoir green bridges for TYLCV to survive between crop seasons. Plant health management approaches include the use of resistant cultivars working on the principle of resistance (*R*) genes, RNA interference (RNAi), and vector management by insecticides, and cultural practices such as adjusting planting date and crop-free period [1, 12–14]. In tomato, host plant resistance to TYLCV was mainly based on the use of *Ty* genes [15]. Nevertheless, the genomic features of TYLCV are altered owing to multiple factors such as virus recombination, genetic mutations, and inclusion of satellite components and invasion of exogenous whitefly species among others [16, 17]. Hence, TYLCV have the ability to overcome the endogenous and genetically engineered disease control measures, and there is a continuous need to develop novel virus management strategies that are effective and durable.

Viral diseases of plants are widespread in Kuwait and are causing significant economic losses reaching up to 95% in many vegetable crops [18]. These crops comprise the majority of greenhouse and open field agriculture in Kuwait. Mosaic, abnormal leaf color, abnormal vein patterns of leaves, mottling in leaves, spotting patterns in leaves, and abnormal leaf shape, leaf curling, and yellowing were observed on plants grown in Al Wafra and Abdally during 2007, 2010, and 2014 seasons [19]. Recent studies showed that TYLCV infection is causing major economic losses in tomato plants, even upto90%. TYLCV has been reported as a major tomato-infecting virus, but it has not been fully characterized at the molecular level and little is known about the genetic diversity of TYLCV populations in Kuwait. Whiteflies are the main vector for TYLCV [20, 21] and a considerable quantum of research was done regarding the management of whitefly vectors with a goal to reduce the impact of begomoviruses [20, 22, 23]. Considering the economic importance of the viral disease in tomato, caused due to TYLCV, and frequent disease outbreaks inflicting severe crop losses [18–21] this study was framed with an objective of characterizing the genome sequences of TYLCV infecting tomato in Kuwait and to perform a comprehensive genomic analysis. We report the molecular characterization of several TYLCV isolates collected from northern and southern Kuwait and the extent of genetic diversity among the isolates characterized herein and reported earlier from Kuwait, the role of virus recombination events, and the inferences from molecular phylogeny with a view to decipher the evolutionary genomics of the virus.

## Materials and methods

### Sample collection

Two commercial tomato farms (Abdally in northern Kuwait, and Al Wafra region in southern Kuwait) were surveyed and leaf samples from plants that displayed symptoms suggestive of TYLCV infection (640 sample) were collected and brought back to Kuwait Institute for Scientific Research, Environmental and Life Science Research Center, (KISR), Safat, 13109, Kuwait and stored at 4ºC until further use. The virus infected samples were collected during 2020 and 2021.

### Total DNA extraction and TYLCV detection

Total genomic DNA was extracted using the CTAB method and tested for the presence of TYLCV by PCR using primer pair PTYc787 (GTTCGATAATGAGCCCAG) and PTYc1121(ATGTAACAGAAACTCATG) [21]. The samples positive for TYLCV were used to sequence the full-length genome which was carried out in Department of Plant Pathology, Washington State University, Pullman, USA following rolling circle amplification (RCA) method. Briefly, the RCA reaction was performed by mixing the following components: genomic DNA (50 ng), 1 μL of exo-resistant random primers (500 μM, 35 optical density units (OU)/mL), 2 μL of 10X reaction buffer, to bring the volume to 10.2 μL using water. The RCA reaction mixture was heated at 95 °C for 5 min in water bath, followed by chilling on ice for 2 min. Following this, 2 μL of 10 mM dNTP, 1.6 μL of phi-29 DNA polymerase, 0.2 μL of pyrophosphate, inorganic (0.1U/μL) were added and incubated at 30 °C for 18 h. The RCA reaction was carried out in a thermal cycler machine. The reaction was stopped by heating the components at 65 °C for 10 min. The RCA derived reaction products were resolved in agarose gel electrophoresis and the amplicons were directly sequenced using the Sanger DNA sequencing protocol.

### In silico analysis

Sequence comparisons of the fifteen Abdally TYLCV isolates, sixteen Al Wafra isolates and three previously reported KISR isolates were performed using the Sequence Demarcation Tool (SDT) [24]. Sequence alignment was generated using ClustalW algorithm. A molecular phylogenetic tree was constructed using the maximum-likelihood method (default parameters with 1000 replicates in the bootstrap analysis) using these sequences along with TYLCV genome sequences available in GenBank utilizing MEGAX [25]. Phylogeny was inferred from using, General Time Reversal (GTR) model with G + I (invariant sites and distributed range) [25]. Potential recombination events were detected by Recombination Detection Program-4 (RDP 4 Beta 4.16) [26]. All the complete TYLCV genome sequences reported from Kuwait and available in GenBank were used in the analysis and the sequence alignment was carried out using ClustalW in MEGA X [25] and the aligned sequences were used for recombination detection studies. For identifying recombination events, step-down correction with the highest acceptable p-value setting of 0.05 was used along with other default settings for all of nine methods (RDP, Chimaera, BootScan, 3Seq, GENECONV, MaxChi, SiScan and LARD, PhylPro) available in the RDP 4 [26].

## Results and discussion

**Fig. 1:**
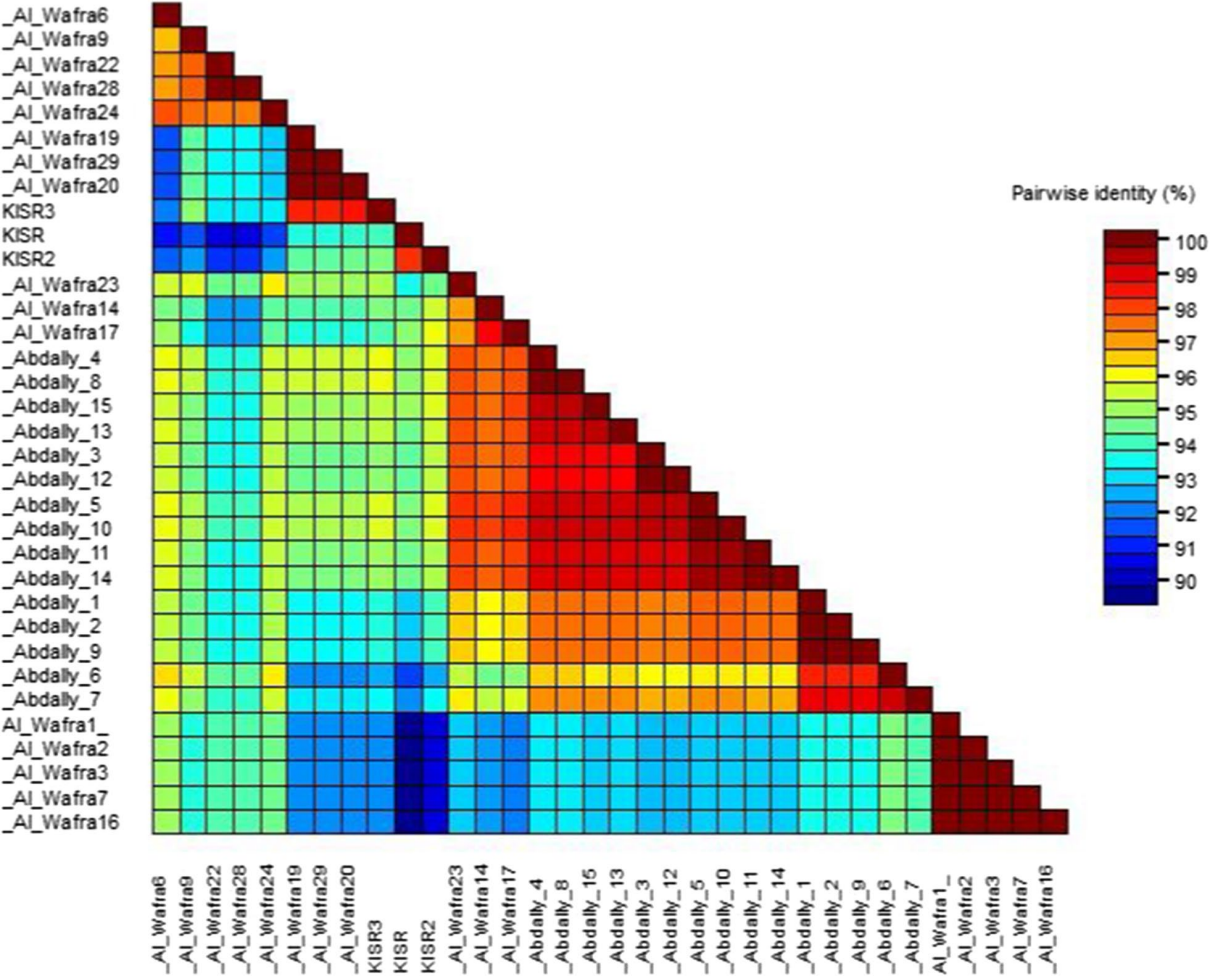
Comparative sequence analysis based on the whole genome sequences of tomato yellow leaf curl virus isolates from two different regions of Kuwait

**Fig. 2:**
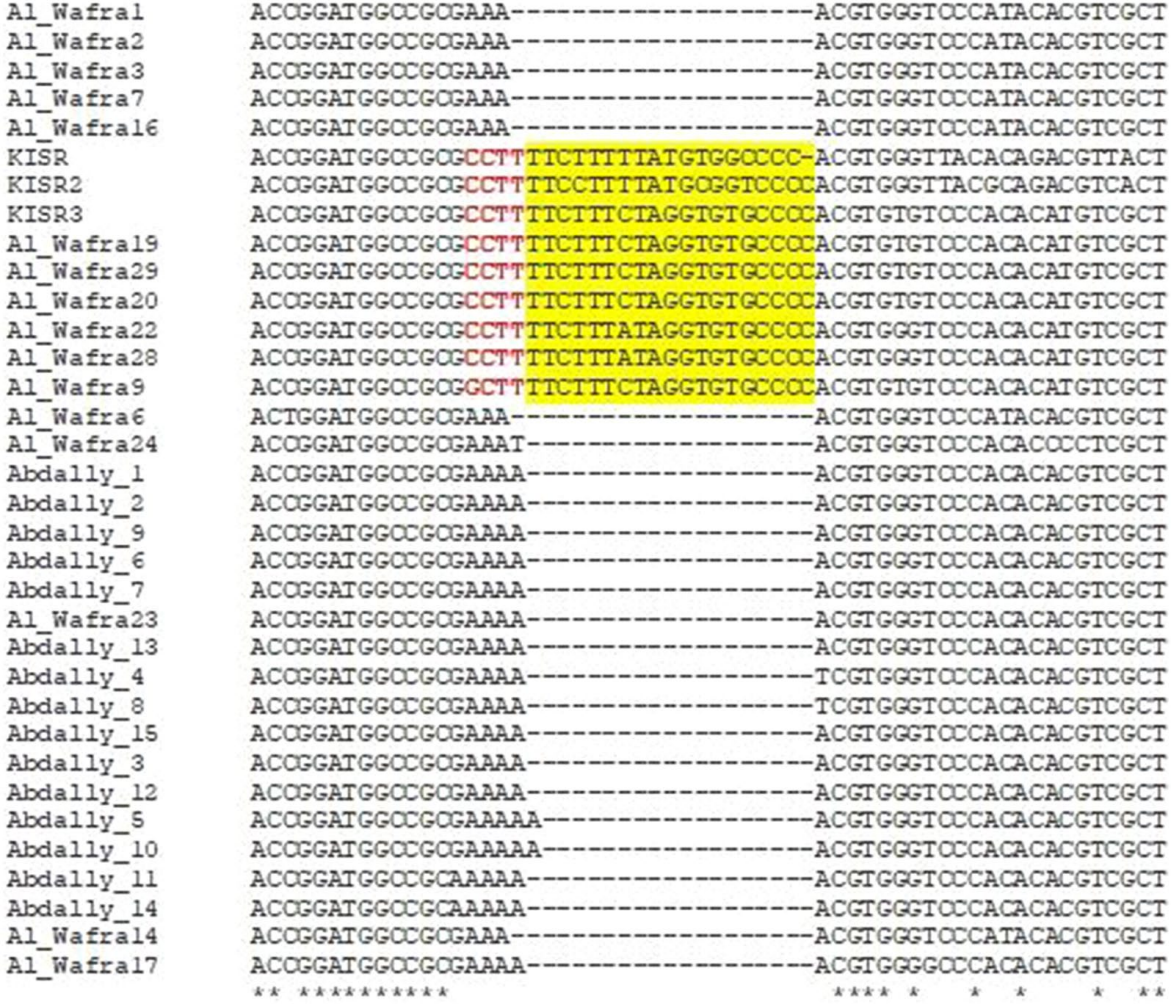
Multiple sequence alignment of all tomato yellow leaf curl virus isolates reported from Kuwait. Three previously reported KISR isolates and six out of sixteen Al Wafra isolates had 19 extra nucleotides (highlighted in yellow) near the 5′-end

Molecular genomic analysis of geminiviruses has been the mainstay in deciphering the genomic diversity of the virus species infecting economically important crops and in devising suitable disease control measures [1, 2, 14]. Hence, in this study, the complete genomic sequences of thirty-one TYLCV isolates from Kuwait were sequenced and deposited in GenBank database (Table 1). The nucleotide identity analysis of various TYLCV genes from these isolates were shown along with the previously reported three isolates (KISR, KISR2 and KISR3) from Kuwait [18, 19] (Fig. 1). The nucleotide sequence identity of Kuwaiti isolates of TYLCV range from 91.20 to 100%. Compared to all fifteen Abdally TYLCV isolates, the three previously reported KISR isolates and six out of sixteen Al Wafra isolates have 19 extra nucleotides (TTCTTTCTAGGTGTGCCCC) in the intergenic region (Fig. 2). There exist 4-nucleotide variations before the 19 extra nucleotides. The three previously reported isolates and six Al Wafra isolates had “G/CCTT” before the 19 extra nucleotides, whereas the others possessed “AAA(A)”.

**Table 1:** List of whole genome sequences of tomato yellow leaf curl virus isolates reported from Kuwait and their NCBI GenBank accession details

No	TYLCV isolates reported from Kuwait	GenBank accession
1	Abdally 1A	OL890666
2	Abdally 2A	OL890667
3	Abdally 4A	OL890668
4	Abdally 6A	OL890669
5	Abdally 3B	OL890670
6	Abdally 5B	OL890671
7	Abdally 7B	OL890672
8	Abdally 8B	OL890673
9	Abdally 9B	OL890674
10	Abdally 10B	OL890675
11	Abdally 11B	OL890676
12	Abdally 12B	OL890677
13	Abdally 13B	OL890678
14	Abdally 14B	OL890679
15	Abdally 15B	OL890680
16	AlWafra1	OM691678
17	AlWafra2	OM691679
18	AlWafra6	OM691680
19	AlWafra7	OM691681
20	AlWafra9	OM691682
21	AlWafra14	OM691683
22	AlWafra16	OM691684
23	AlWafra17	OM691685
24	AlWafra19	OM691686
25	AlWafra20	OM691687
26	AlWafra22	OM691688
27	AlWafra23	OM691689
28	AlWafra24	OM691690
29	AlWafra28	OM691691
30	AlWafra29	OM691692

**Fig. 3:**
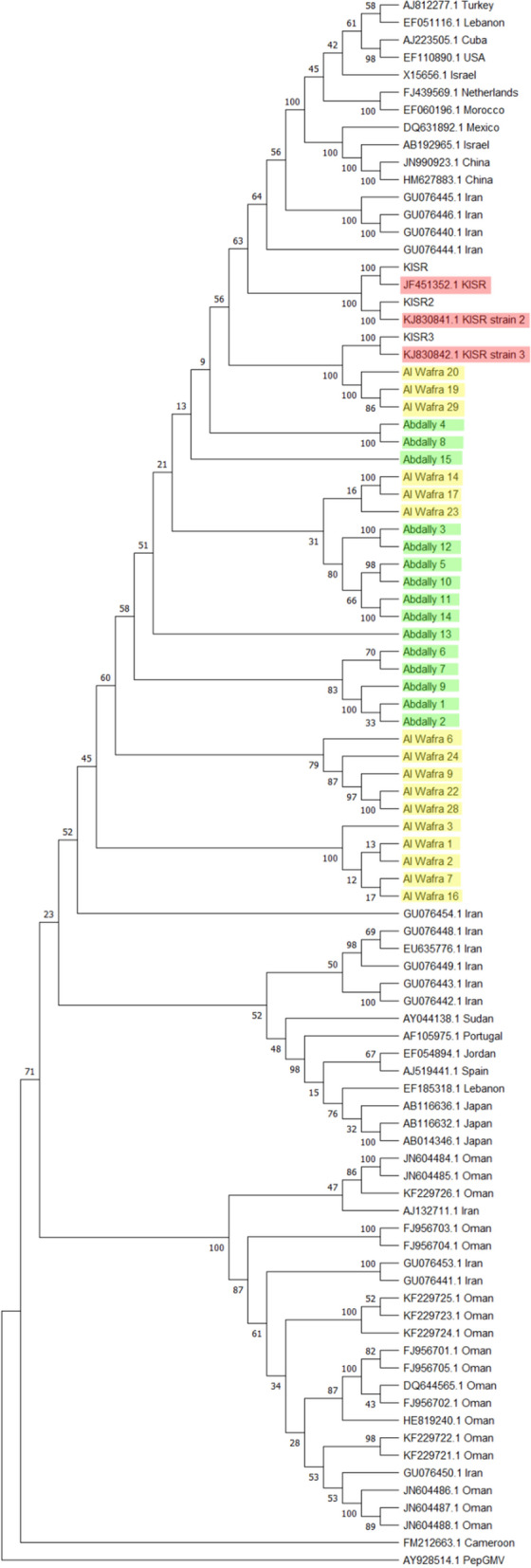
Phylogenetic tree based on the whole genome sequences of Tomato yellow leaf curl virus isolates reported from Kuwait and other parts of world. Evolutionary history was inferred by using Maximum Likelihood method and Tamura-Nei Model. The phylogenetic tree was rooted to Pepper golden mosaic virus isolate (AY928514). Three previously reported Kuwaiti isolates are shown in red boxes. The sequences of AI Wafra isolates are highlighted in yellow and Abdally isolates are highlighted in green

Phylogenetic analyses were conducted to compare complete nucleotide sequences with those from other parts of the world and to infer molecular evolution of current isolates (Fig. 3). A total of eighty-seven TYLCV isolates were analyzed, which formed two distinct clades. All 34 Kuwaiti isolates (31 from this study and three previously reported) grouped into a clade along with the isolates from China, Iran, Israel, Japan, Jordan, Mexico, Oman, Portugal, Turkey and the US (Fig. 3). TYLCV isolates reported from Cuba, Japan, Lebanon, Morocco, The Netherlands, and Spain formed a distinct clade (Fig. 3). Earlier study on molecular analysis of TYLCV in the Arabian Peninsula and Iran suggests these regions as the centre of diversity [11].

**Fig. 4:**
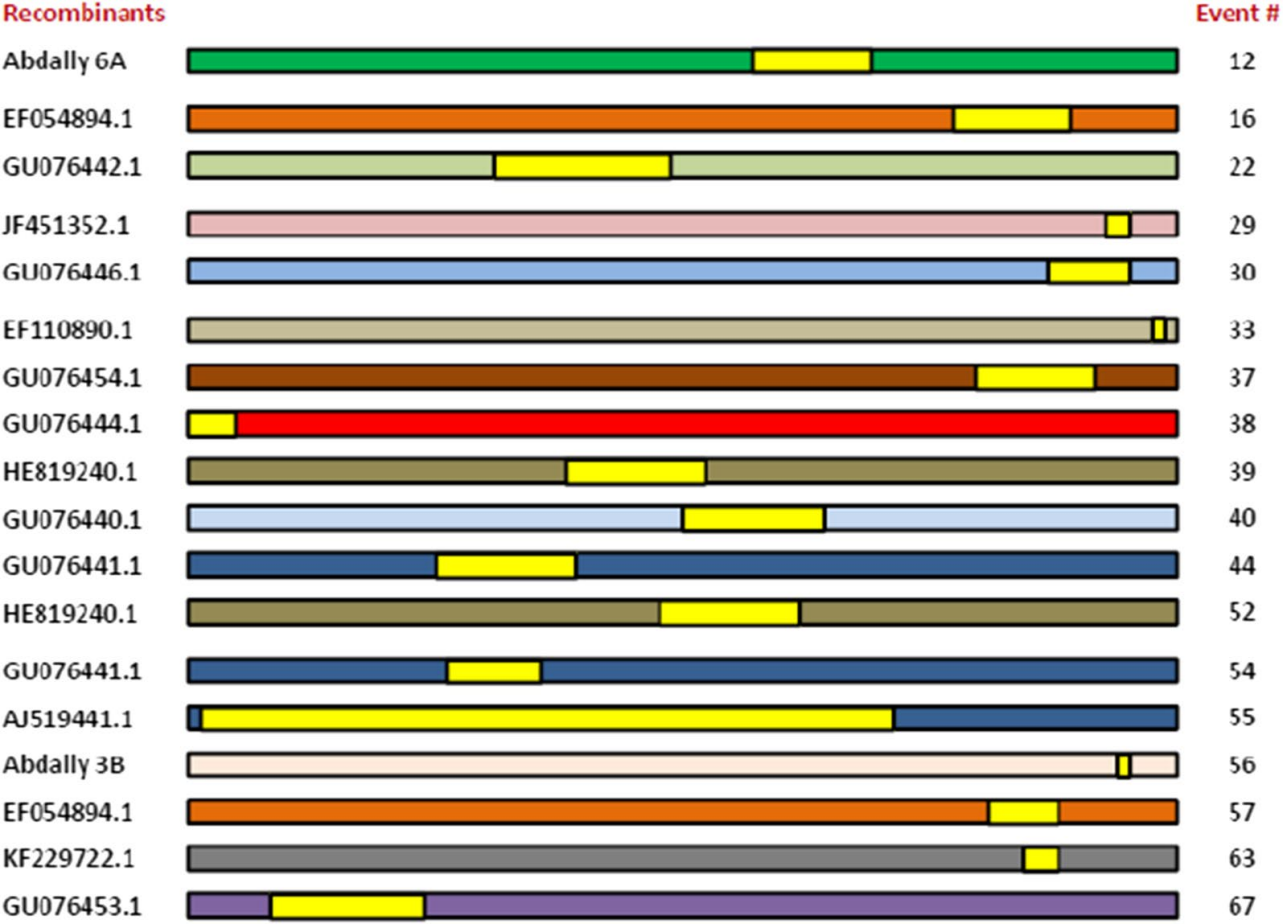
Recombinants detected by Recombination Detection Program (RDP v 4) among various tomato yellow leaf curl virus isolates. The event numbers are shown. (details of the major and minor parents and *p*-values are provided in Table 2)

To identify putative recombination events, an analysis was performed using the RDP4 program. Fourteen recombination events in the 87 TYLCV full-length sequences from worldwide were detected by at least four methods in the RDP4 program (Table 2). The isolates Abdally 6A (OL890669) and Abdally 3B (OL890670) reported in this study were identified to be potential recombinants. In addition, TYLCV isolates Abdally 11B (OL890676), Abdally 13B (OL890678), and Abdally 3B (OL890670) served as major parents for generation of different recombinants. It suggests the occurrence of microevolution within the TYLCV populations in Kuwait (Table 2 and Fig. 4). Further, genetic recombinant TYLCV isolates were shown to have ecologically selective advantage over their parental viruses [17].

**Table 2:** Potential genetic recombination events identified in tomato yellow leaf curl virus genomes as detected by Recombination Detection Program (RDP)

Potential recombinant	Major parent	Minor parent	Recombination detection methods	Recombination event #	*P*-value
Abdally6A (OL890669)	Abdally 11B ( OL890676)	EU635776.1	RDP, GENECONV, BootScan, MaxChi, Chimaera, SiScan, 3Seq	12	8.578 E−30
EF054894.1	KJ830842.1	GU076444.1	RDP, GENECONV, BootScan, MaxChi, SiScan, 3Seq	16	6.075 E−26
GU076442.1	KF229722.1	GU076449.1	RDP, GENECONV, BootScan, MaxChi, Chimaera, SiScan, 3Seq	22	8.755 E−23
JF451352.1	AF105975.1	AJ223505.1	RDP, GENECONV, MaxChi, Chimaera	29	1.701 E−16
GU076446.1	KJ830842.1	EF054894.1	RDP, GENECONV, MaxChi, Chimaera, SiScan, 3Seq	30	1.474 E−15
EF110890.1	JN990923.1	FJ439569.1	RDP, GENECONV, SiScan, 3Seq	33	9.571 E−14
GU076454.1	KF229721.1	GU076449.1	RDP, GENECONV, BootScan, MaxChi, SiScan, 3Seq	37	4.990 E−12
GU076444.1	KF229721.1	EU635776.1	RDP, GENECONV, MaxChi, Chimaera, SiScan, 3Seq	38	9.783 E−13
HE819240.1	GU076445.1	Abdally 3B (OL890670)	RDP, GENECONV, 3Seq	39	6.449 E−11
GU076440.1	HE819240.1	HM627883.1	RDP, GENECONV, BootScan, MaxChi, SiScan,	40	9.140 E−09
GU076441.1	TYLCV-13B RCA	GU076440.1	GENECONV, Chimaera, SiScan, 3Seq	44	6.548 E−13
HE819240.1	KJ830842.1	GU076440.1	RDP, GENECONV, MaxChi, Chimaera, SiScan, 3Seq	52	3.072 E−06
GU076441.1	KF229722.1	Abdally 13B ( OL890678)	RDP, GENECONV, BootScan, MaxChi, Chimaera, SiScan, 3Seq	54	4.634 E−06
AJ519441.1	AJ223505.1	FJ439569.1	RDP, MaxChi, Chimaera, SiScan, 3Seq	55	7.900 E−07
Abdally 3B (OL890670)	EU635776.1	EF060196.1	RDP, GENECONV	56	2.381 E−04
EF054894.1	EF110890.1	AJ132711.1	RDP, GENECONV, BootScan, MaxChi, Chimaera, SiScan, 3Seq	57	1.542 E-03
KF229722.1	GU076449.1	AJ132711.1	GENECONV, MaxChi, SiScan	63	1.032 E−12
GU076453.1	Abdally 3B (OL890670)	GU076454.1	MaxChi, Chimaera	67	1.050 E−02

Comparative sequence analysis of Kuwaiti TYLCV isolates reveal > 90% sequence identity, suggesting that these isolates may have derived from a few parental strains originally imported into the country (Fig. 1). The additional 19 nucleotides observed in nine Kuwaiti isolates indicate that these isolates might have resulted from a single gene recombination/insertion event. The further integration of “CCTT” motif could represent a second mutation event occurred at a later time (Fig. 2). The phylogenetic analysis of all known TYLCV sequences reported worldwide suggests that TYLCV seems to undergo a relatively rapid evolution and exhibit significant genetic variation. The isolates Al Wafra 1, Al Wafra 7, Al Wafra2, AlWafra 16, Al Wafra 3, and Al Wafra 6 were genetically distinct and formed a separated cluster. However, some TYLCV isolates viz., Al Wafra 17, Al Wafra 23, Al Wafra 24 and Abdally 15 shared phylogenetic lineage with TYLCV isolates reported from Iran and Oman (Fig. 3). On the other hand, all the Abdally isolates, excluding Abdally 15, showed monophyletic origin along with TYLCV isolates reported from Oman (Fig. 3).The phylogenetic relatedness of Kuwait TYLCV isolates with that of Iran and Oman imply the cross border transfer or movement of virus isolates among the countries in Persian Gulf region (Fig. 3). If the observed diversity in sequences in selected isolates has any role in modulating the TYLCV-tomato interactions remains to be seen and the current study lays groundwork for further investigations.

In conclusion, we report here the complete genome sequences of 31 isolates of TYLCV circulating in the tomato fields of Northern and Southern Kuwait. A comprehensive comparative genomic analysis of the novel TYLCV isolates along with those reported earlier identified recombination events that could possibly involve in the evolution of TYLCV causing the generation of novel variants of concern. The information presented in this article will be quite useful for the comprehension of TYLCV biology, epidemiology and disease control measures.

## Limitations

No limitations were encountered during the study. Our findings form the basis for further studies on the functional significance of the extra 19 nucleotides found in the 5′ end of genomic sequences of KISR and six Al Wafra isolates.

## Data Availability

The viral genome sequence datasets generated and described in this research are deposited in the NCBI GenBank database (ncbi.nlm.nih.gov) and are available under accession numbers (Al-Wafra isolates: OM691678 to OM691692) and (Al-Abdally isolates: OL890666 to OL890680).The data described can be freely and openly accessed from the repository.
